# Clinicopathological features and prognostic validity of the European Neuroendocrine Tumor Society (ENETS) and American Joint Committee on Cancer (AJCC) 8th staging systems in colonic neuroendocrine neoplasms

**DOI:** 10.1002/cam4.2370

**Published:** 2019-07-10

**Authors:** Yu Zhang, Liang Shang, Pan‐Pan Zhang, Luo‐Hai Chen, Wei Wang, Cheng Fang, Meng Qiu, Xing‐Yu Feng, Lin Zhou, Meng Zhang, Huang‐Ying Tan, Xu‐Dong Qiu, Hao Wang, Rong Lin, Qin Zhang, Yu‐Jie Zeng, Kai‐Zhou Jin, Xian‐Jun Yu, Lin Shen, Min‐Hu Chen, Jie Li, Le‐Ping Li, Jie Chen

**Affiliations:** ^1^ Department of Gastroenterology The First Affiliated Hospital Sun Yat‐sen University Guangzhou China; ^2^ Department of Gastrointestinal Surgery Shandong Provincial Hospital Affiliated to Shandong University Jinan China; ^3^ Department of Gastrointestinal Oncology Key laboratory of Carcinogenesis and Translational Research (Ministry of Education) Peking University Cancer Hospital & Institute Beijing China; ^4^ Department of Gastric Surgery Sun Yat‐sen University Cancer Center Guangzhou China; ^5^ Division of Abdominal Cancer Cancer Center West China Hospital of Sichuan University Chengdu China; ^6^ Department of General Surgery Guangdong General Hospital Guangzhou China; ^7^ Department of Gastroenterology The First Affiliated Hospital of Zhengzhou University Zhengzhou China; ^8^ Department of Integrative Oncology China‐Japan Friendship Hospital Beijing China; ^9^ Department of Colorectal Surgery Changhai Hospital Second Military Medical University Shanghai China; ^10^ Division of Gastroenterology Union Hospital Tongji Medical College Huazhong University of Science and Technology Wuhan China; ^11^ Division of Pathology Union Hospital Tongji Medical College Huazhong University of Science and Technology Wuhan China; ^12^ Department of Gastroenteropancreatic Surgery Sun Yat‐sen Memorial Hospital Sun Yat‐sen University Guangzhou China; ^13^ Department of Pancreatic Surgery Fudan University Shanghai Cancer Center Shanghai China

**Keywords:** clinicopathological features, colon, neuroendocrine neoplasms, tumor stage

## Abstract

**Purpose:**

This study aimed to investigate the characteristics of colonic neuroendocrine neoplasms (NENs) and to validate the prognostic value of the European Neuroendocrine Tumor Society (ENETS) and American Joint Committee on Cancer (AJCC) 8th staging systems.

**Methods:**

A total of 167 and 1248 patients with colonic NENs from 12 medical centers across China and from the Surveillance, Epidemiology, and End Results (SEER) cancer registry in the United States, respectively, were reviewed. Patients were staged according to the ENETS and AJCC 8th staging systems.

**Results:**

Clinicopathological features of colonic NENs in the Chinese cohort and SEER cohort were significantly distinct. In both the Chinese cohort and the SEER cohort, colonic neuroendocrine carcinoma (NEC) and mixed adeno‐neuroendocrine carcinoma (MANEC) were more frequent in the midgut than in the hindgut. Tumors originating from the midgut tended to be larger and at a more advanced stage than those from the hindgut. The AJCC 8th staging system and the ENETS system appeared to have similar prognostic ability for colonic NEC/MANEC.

**Conclusions:**

Our study revealed that tumors originating from the midgut and the hindgut shared different clinicopathological features. The AJCC 8th staging system and the ENETS system appeared to have similar prognostic ability for colonic NEC/MANEC.

## 1 INTRODUCTION

Neuroendocrine neoplasms (NENs) are rare neoplasms with great heterogeneity that originate from peptidergic neurons and neuroendocrine cells throughout the body. According to the embryonic origin of the site of tumor onset, NENs are classified as foregut, midgut or hindgut NENs. The foregut includes the digestive tract from the oropharynx to the upper duodenum, liver, gallbladder, pancreas and the respiratory system below the throat; the midgut includes the middle part of the duodenum to the right two‐thirds of the transverse colon; and the hindgut begins at the left one‐third of the transverse colon and goes to the upper anal canal.^1^ In addition, according to tumor differentiation, NENs include well or moderately differentiated neuroendocrine tumor (NET), poorly differentiated neuroendocrine carcinoma (NEC) and mixed adeno‐neuroendocrine carcinoma (MANEC). The biological behavior of NET is relatively benign compared with NEC/MANEC. The incidence of NENs has been rising in recent decades.^2^, ^3^ Colonic NENs, as a rare kind of NENs originating from the cecum to the sigmoid colon, account for just 4%‐8% of all NENs.^3^, ^4^, ^5^, ^6^, ^7^, ^8^ Most of the previous studies combined colonic NENs with rectal NENs, and the few studies that focused on colonic NENs had small sample sizes.^9^, ^10^, ^11^ However, with increasing incidence and detection rates, clinicians noticed significant differences between colonic NENs and rectal NENs.^12^ Therefore, it is necessary to investigate them separately. Given the rarity of colonic NENs, a population‐based study is urgently needed to provide an overview of the clinicopathological features and survival of this rare subtype of NENs. The primary aim of the current study was to investigate the clinicopathological features of patients with colonic NENs.

In 2007, the European Neuroendocrine Tumor Society (ENETS) first proposed a formal TNM system for colorectal neuroendocrine neoplasms (Appendices),^13^ which was then adopted by the American Joint Committee on Cancer (AJCC) for the 7th edition of its staging manual in 2010.^14^ The AJCC published the 8th edition of the staging manual in 2016 (Appendices);^15^ in this new version, guidelines for colonic NEC/MANEC followed those for colonic adenocarcinoma, while guidelines for well or moderately differentiated colonic NET remained the same as in the ENETS system. However, no studies have compared the prognostic validity of the ENETS and AJCC 8th staging system in colonic NEC/MANEC. Thus, the second aim of the current study was to validate the prognostic value of the ENETS and AJCC 8th staging systems for colonic NENs.

## 2 METHODS

### 2.1 Patients and data collection

We collected multicentric data from China retrospectively. Clinical data of patients with pathologically confirmed colonic NENs from 1 January 2000 to 26 August 2017 were retrieved from 12 Chinese medical centers including The First Affiliated Hospital, Sun Yat‐Sen University (n = 19), Shandong Provincial Hospital Affiliated to Shandong University (n = 38), Peking University Cancer Hospital & Institute (n = 35), Sun Yat‐sen University Cancer Center (n = 15), West China Hospital of Sichuan University (n = 13), Guangdong General Hospital (n = 10), The First Affiliated Hospital of Zhengzhou University (n = 10), China‐Japan Friendship Hospital (n = 9), Changhai Hospital, Second Military Medical University (n = 7), Union Hospital, Tongji Medical College, Huazhong University of Science and Technology (n = 6), Sun Yat‐sen Memorial Hospital of Sun Yat‐Sen University (n = 3), and Fudan University Shanghai Cancer Center (n = 2). These twelve hospitals were located in the north, central, east, west and south of China; all of them were representative general hospitals or cancer centers in their regions, and they were all members of the Chinese Study Group for Neuroendocrine Tumors. Patients who had other previous or concomitant kinds of cancer or documented familial NENs such as multiple endocrine neoplasia type 1, were excluded, and rectal NENs were excluded. The current study was approved by the ethics committees of the twelve hospitals, and consent was obtained from all patients.

We retrieved data of patients with colonic NENs from the SEER database in the US for comparison and validation. Data were retrieved with SEER*Stat software (version 8.3.4, 22 March 2017; Cancer Statistics Branch, NCI, Bethesda, MD). Because of the unidentifiable patient information in the SEER database, this study was exempted for approval by the Office of Human Subjects Research of the National Institutes of Health. The following International Classification of Disease for Oncology, Third Revision (ICD‐O‐3) codes were applied to identify colonic NENs: 8013 and 8240‐8249. In order, these were 8013, large cell neuroendocrine carcinoma; 8240, carcinoid tumor; 8241, enterochromaffin; 8242, enterochromaffin‐like; 8243, goblet; 8244, mixed adeno‐neuroendocrine carcinoma; 8245, adenocarcinoid; 8246, neuroendocrine carcinoma; and 8249, atypical carcinoid. We selected NENs with a primary site of colon (site code: C18.0, C18.2‐18.9), while NENs with a primary site of rectum were not included; 18.0, cecum; 18.2, ascending colon; 18.3, hepatic flexure; 18.4, transverse colon; 18.5, splenic flexure; 18.6, descending colon; 18.7, sigmoid colon; 18.8, overlapping lesions of colon; 18.9, colon, not otherwise specified. Only patients diagnosed with positive pathology from 2000 to 2014 were included. Patients who had a history of other cancers or diagnosed at autopsy or death certificate were excluded.

Data including age at diagnosis, sex, date of initial diagnosis, embryonic origin of primary tumor, tumor differentiation, tumor size and invasion, nodal status, distant metastasis, ENETS stage, AJCC 8th stage and follow‐up data were retrieved from both the Chinese cohort and SEER database. In addition, tumor grade according to the WHO 2010 classification based on Ki‐67 index and mitotic count was available in the Chinese cohort.^16^ In the SEER cohort, tumor grade was not available, and only tumor differentiation was collected. All data were reviewed and checked independently by Yu Zhang and Luohai Chen.

### 2.2 Grading and staging classification systems

Tumor grade was determined by the Ki‐67 index and mitotic count according to the WHO 2010 classification in the Chinese cohort. The Ki‐67 index was detected using MIB‐1 antibody and counted in areas with the strongest nuclear labeling. Mitotic count was evaluated at least 50 high power fields (HPFs) (1HPF = 0.2 mm^2^). When discrepancy between the Ki‐67 index and mitotic count occurred, the measurement indicating a higher grade was applied. Three grades were given according to the WHO 2010 classification: Grade 1 (G1, Ki‐67 index ≤2% and/or mitotic count <2/10HPF), Grade 2 (G2, Ki‐67 index: 3%‐20% and/or mitotic count: 2‐20/10HPF), Grade 3 (G3, Ki‐67index >20% and/or mitotic count >20/10HPF). All pathological sections were reviewed by specified expert pathologists from the included hospitals. Tumor stages were determined according to the ENETS and AJCC 8th staging systems.

### 2.3 Statistical analysis

SPSS statistical software version 20.0 (SPSS Inc Chicago, IL) was used to analyze the data. Categorical variables (ie, embryonic origin of tumor, tumor invasion, and distant metastasis) were grouped for clinical reasons. Categorical variables were compared using the chi‐squared test when the expected values were five or more in at least 80% of the cells and no cell had an expected value of <1, or Fisher's exact test when the data set was too small to meet the sample size assumption of the chi‐squared test in 2 × 2 tables. For two groups with continuous variables, the *t* test was used when each set of data met the normal distribution and the homogeneity of variance; otherwise, the Mann‐Whitney U test was used. For multiple groups with continuous variables, one‐way analysis of variance (ANOVA) was used when the data met the normal distribution and the homogeneity of variance; otherwise, the Kruskal‐Wallis H test was used. The Bonferroni method was used to adjust *P* values when making pairwise comparisons. Survival time was calculated from the date of initial diagnosis until the date of death or last follow‐up. Kaplan‐Meier analysis with the log‐rank test was performed to analyze disease‐specific survival (DSS). The staging system efficacy for predicting outcome was evaluated by calculating the concordance index (C‐index), which ranges from 0.5 to 1.0, with 0.5 indicating a random chance and 1.0 indicating a perfect discrimination ability. Statistical significance was defined as two‐sided *P* < 0.05.

## 3 RESULTS

### 3.1 Patients' characteristics

In total, 167 and 1248 patients with colonic NENs from the Chinese hospitals and the SEER database were included, respectively (Table 1). Many characteristics of patients at baseline were significantly different between the two cohorts. The median age of patients in the Chinese and SEER cohorts was 60 (range, 14‐85) and 60 (range, 15‐95), respectively, and the proportion of male patients was 59.9% (n = 100) and 48.8% (n = 609) in the two groups, respectively (*P* = 0.007). In the Chinese and SEER cohorts, 53.8% (n = 85) and 66.7% (n = 725), respectively, of colonic NENs originated from the midgut (*P* = 0.001). A total of 74.9% (n = 125) of Chinese patients and 43.6% (n = 267) of patients from the SEER cohort had poorly differentiated NEC/ MANEC (*P* < 0.001). In addition, tumor size of colonic NENs in Chinese patients and in patients from the SEER cohort were 4.1 and 3.0 cm, respectively (*P* < 0.001). Lymph node metastasis (LNM) was found in 59.7% (n = 95) of Chinese patients and 55.5% (n = 595) of patients from the SEER cohort. Distant metastasis was found in 43.0% (n = 71) of Chinese patients and 32.0% (n = 391) of patients from the SEER cohort (*P* = 0.005). In total, 74.4% (n = 119) of Chinese patients and 77.6% (n = 755) of patients from the SEER cohort had stage III/IV disease according to the ENETS staging system. A total of 68.1% (n = 109) of Chinese patients and 80.8% (n = 430) of patients from the SEER cohort had stage III/IV disease according to the AJCC 8th staging system (*P* = 0.001).

**Table 1:** Clinical features of colonic NENs at baseline

Characteristics	Chinese patients (N = 167)	SEER database (N = 1248)	*P*
Age (y)			
Mean	58.0 ± 14.7	61.2 ± 13.5	0.058
Median	60.0	60.0	
Sex			
Male	100 (59.9%)	609 (48.8%)	0.007
Female	67 (40.1%)	639 (51.2%)	
Embryonic origin^a^			
Midgut	85 (53.8%)	725 (66.7%)	0.001
Hindgut	73 (46.2%)	362 (33.3%)	
Tumor grade^b^			
Grade 1	32 (19.2%)		
Grade 2	8 (4.8%)		
Grade 3	127 (76.0%)		
Tumor differentiation^c^			
NET	42 (25.1%)	345 (56.4%)	<0.001
NEC/MANEC	125 (74.9%)	267 (43.6%)	
Tumor size^d^			
Mean (cm)	4.8 ± 3.2	3.7 ± 2.9	<0.001
Median (cm)	4.1	3.0	
Tumor invasion^e^			
Submucosa	25 (17.6%)	257 (30.1%)	<0.001
Subserosa	69 (48.6%)	277 (32.4%)	
Peritoneum/other organ	48 (33.8%)	321 (37.5%)	
Lymph node metastasis^f^			
No	64 (40.3%)	478 (44.5%)	0.308
Yes	95 (59.7%)	595 (55.5%)	
Distant metastasis^g^			
No	94 (57.0%)	832 (68.0%)	0.005
Yes	71 (43.0%)	391 (32.0%)	
ENETS stage^h^			
I/II	41 (25.6%)	218 (22.4%)	0.369
III/IV	119 (74.4%)	755 (77.6%)	
AJCC 8^h^ stage^i^			
I/II	51 (31.9%)	102 (19.2%)	0.001
III/IV	109 (68.1%)	430 (80.8%)	

Abbreviations: AJCC, American Joint Committee on Cancer; ENETS, European Neuroendocrine Tumor Society; MANEC, Mixed adeno‐neuroendocrine carcinoma; NEC, neuroendocrine carcinoma; NENs, Neuroendocrine neoplasms; SEER, Surveillance, Epidemiology, and End Results.

a Data were available for 158 Chinese patients and 1087 patients from SEER database.

b Data were not available for patients from SEER database.

c Data were available for 167 Chinese patients and 612 patients from SEER database.

d Data were available for 136 Chinese patients and 787 patients from SEER database.

e Data were available for 142 Chinese patients and 855 patients from SEER database.

f Data were available for 159 Chinese patients and 1073 patients from SEER database.

g Data were available for 165 Chinese patients and 1223 from SEER database.

h Data were available for 160 Chinese patients and 973 from SEER database.

i Data were available for 160 Chinese patients and 532 from SEER database.

### 3.2 Comparison of the clinical features of colonic NENs in the midgut and hindgut

Since the biological behavior of colonic adenocarcinoma located in the left‐sided colon and the right‐sided colon, which originate from the hindgut and midgut, respectively, are significantly distinct,^17^, ^18^, ^19^, ^20^ we further compared the clinical features of colonic NENs between the midgut and hindgut (Table 2). In the Chinese cohort, 158 out of 167 patients in the cohort were included for analyses, since the tumor origin of nine patients was unknown. Colonic NEC/MANEC was more common in the midgut than in the hindgut (87.1% vs 58.9%, *P* < 0.001). The median tumor size of colonic NENs in the midgut was significantly larger than that in the hindgut (5.0 vs 3.5 cm, *P* < 0.001). In addition, tumors with peritoneum or other organ invasion were more common in the midgut than in the hindgut (46.5% vs 14.3%, *P* < 0.001). Tumors with LNM were more common in the midgut than in the hindgut (66.7% vs 50.0%, *P* = 0.038). Patients with stage III/IV disease according to both the ENETS and AJCC 8th staging systems had a significantly larger proportion in the midgut than in the hindgut (86.4% vs 57.1%, *P* < 0.001; 79.0% vs 52.9%, *P* = 0.001, respectively).

**Table 2:** Comparison of the clinical features between colonic NENs in the midgut and hindgut

Characteristics	Chinese patients (N = 158)			Patients in SEER database (N = 1087)		
	Midgut (N = 85)	Hindgut (N = 73)	*P*	Midgut (N = 725)	Hindgut (N = 362)	*P*
Age (y)						
Mean	60.1 ± 15.5	56.4 ± 13.8	0.053	62.9 ± 13.3	57.2 ± 13.0	<0.001
Median	62.0	59.0		63.0	56.0	
Sex						
Male	54 (63.5%)	41 (56.2%)	0.346	331 (45.7%)	198 (54.7%)	0.005
Female	31 (36.5%)	32 (44.8%)		394 (54.3%)	164 (45.3%)	
Tumor differentiation^a^						
NET	11 (12.9%)	30 (41.1%)	<0.001	225 (54.9%)	93 (66.9%)	0.013
NEC/MANEC	74 (87.1%)	43 (58.9%)		185 (45.1%)	46 (33.1%)	
Tumor size (cm)^b^						
Mean	5.7 ± 3.1	3.6 ± 3.0	<0.001	3.9 ± 2.8	2.3 ± 2.8	<0.001
Median	5.0	3.5		3.5	1.0	
Tumor invasion^c^						
Submucosa	2 (2.8%)	23 (36.5%)	<0.001	54 (9.9%)	179 (74.6%)	<0.001
Subserosa	36 (50.7%)	31 (49.2%)		234 (42.7%)	30 (12.5%)	
Peritoneum/other organ	33 (46.5%)	9 (14.3%)		260 (47.4%)	31 (12.9%)	
Lymph node metastasis^d^						
No	27 (33.3%)	35 (50.0%)	0.038	179 (26.6%)	239 (80.7%)	<0.001
Yes	54 (66.7%)	35 (50.0%)		494 (73.4%)	57 (19.3%)	
Distant metastasis^e^						
No	43 (51.2%)	48 (66.7%)	0.051	433 (60.5%)	308 (86.5%)	<0.001
Yes	41 (48.8%)	24 (33.3%)		283 (39.5%)	48 (13.5%)	
ENETS stage^f^						
I/II	11 (13.6%)	30 (42.9%)	<0.001	86 (12.7%)	120 (60.3%)	<0.001
III/IV	70 (86.4%)	40 (57.1%)		593 (87.3%)	79 (39.7%)	
AJCC 8th stage^g^						
I/II	17 (21.0%)	33 (47.1%)	0.001	57 (14.4%)	41 (44.6%)	<0.001
III/IV	64 (79.0%)	37 (52.9%)		339 (85.6%)	51 (55.4%)	

Abbreviations: AJCC, American Joint Committee on Cancer; ENETS, European Neuroendocrine Tumor Society; MANEC, Mixed adeno‐neuroendocrine carcinoma; NEC, neuroendocrine carcinoma; NENs, Neuroendocrine neoplasms; NOS, Not otherwise specified; SEER, Surveillance, Epidemiology, and End Results.

a Data were available for 158 Chinese patients and 549 patients from SEER database.

b Data were available for 129 Chinese patients and 738 patients from SEER database.

c Data were available for 134 Chinese patients and 788 patients from SEER database.

d Data were available for 151 Chinese patients and 969 patients from SEER database.

e Data were available for 156 Chinese patients and 1072 patients from SEER database.

f Data were available for 151 Chinese patients and 878 patients from SEER database.

g Data were available for 151 Chinese patients and 488 patients from SEER database.

To verify our findings obtained from the Chinese cohort, we further performed analyses in the SEER cohort, and similar results were found (Table 2). One thousand and eighty seven out of 1248 patients in the SEER cohort were included for analyses, since the tumor origin of 161 patients was unknown. Patients with tumors in the midgut were much older than patients with tumors in the hindgut (63 vs 56, *P* < 0.001); the proportion of male patients with tumors in the midgut was lower than in the hindgut (45.7% vs 54.7%, *P* = 0.005). The percentage with colonic NEC/MANEC in the midgut was also significantly higher than that in the hindgut. The median tumor size was significantly larger, and tumors with peritoneum or other organ invasion were also more common in the midgut than in the hindgut. Furthermore, tumors with LNM, distant metastasis and stage III/IV disease according to both the ENETS and AJCC 8th staging systems were also more common in the midgut than in the hindgut.

### 3.3 Survival of patients and prognostic validity of the ENETS and AJCC 8th staging systems

**Figure 1:**
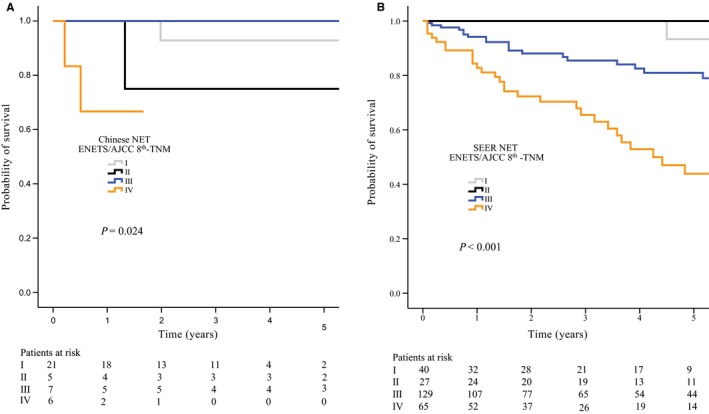
Kaplan‐Meier analysis of Chinese patients with well/moderately differentiated colonic NET according to both the ENETS and AJCC 8th staging systems (A), and Kaplan‐Meier analysis of patients with well/moderately differentiated colonic NET from the SEER database according to both the ENETS and AJCC 8th staging systems (B). Discrepancy between the numbers of patients at risk and the total size of the analytic cohort for each staging system is accounted for by the number of patients who were lost to follow‐up within 30 days. AJCC, American Joint Committee on Cancer; ENETS, European Neuroendocrine Tumor Society; NET, neuroendocrine tumor; SEER, Surveillance, Epidemiology, and End Results

The ENETS and AJCC 8th staging systems shared the same guidelines for well/moderately differentiated colonic NET, and the TNM stage of patients with colonic NET according to the two systems is shown in Table 3. For the 42 Chinese patients with colonic NET, the median follow‐up time was 2.62 years (range, 0.02‐8.54 years), and the 3‐year DSS rates were 92.9%, 75.0%, 100%, and 66.7%, for stages I‐IV, respectively (Figure 1A).Survival comparisons were statistically significant when comparing stages (*P* = 0.024), and the C‐index was 0.853 (95% CI, 0.722‐0.984). For the 345 patients with colonic NET in the SEER cohort, the median follow‐up time was 2.67 years (range, 0.08‐14.0 years), and the 3‐year DSS rates were 100%,100%, 85.5%, and 65.5%, for stages I‐IV, respectively (Figure 1B). Survival comparisons were also statistically significant when comparing stages (*P* < 0.001); the C‐index was 0.722 (95% CI, 0.666‐0.778).

**Table 3:** Stage of well/moderately differentiated colonic NET based on the ENETS and AJCC 8th staging systems

Factor	Chinese cohort	SEER cohort
T classification^a^		
T1	22 (55.0%)	48 (18.9%)
T2	4 (10.0%)	40 (15.7%)
T3	12 (30.0%)	122 (48.0%)
T4	2 (5.0%)	44 (17.3%)
N classification^b^		
N0	27 (69.2%)	126 (39.7%)
N1	12 (30.8%)	191 (60.3%)
M classification^c^		
M0	36 (85.7%)	270 (78.9%)
M1	6 (14.3%)	72 (21.1%)
TNM stage^d^		
I	22 (53.7%)	41 (14.9%)
II	6 (14.6%)	29 (10.5%)
III	7 (17.1%)	134 (48.7%)
IV	6 (14.6%)	71 (25.8%)

Abbreviations: AJCC, American Joint Committee on Cancer; ENETS, European Neuroendocrine Tumor Society; NET, Neuroendocrine tumor; SEER, Surveillance, Epidemiology, and End Results.

a Data were available for 40 Chinese patients and 254 patients from SEER database.

b Data were available for 39 Chinese patients and 317 patients from SEER database.

c Data were available for 42 Chinese patients and 342 patients from SEER database.

d Data were available for 41 Chinese patients and 275 patients from SEER database.

**Figure 2:**
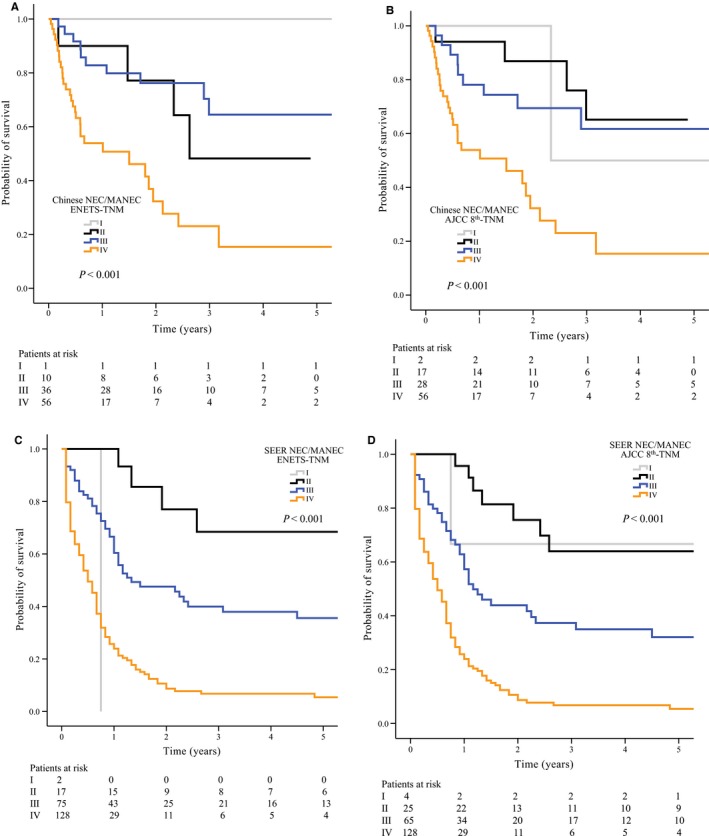
Kaplan‐Meier analysis of Chinese patients with colonic NEC/MANEC according to the ENETS staging system (A), AJCC 8th staging system (B), and Kaplan‐Meier analysis of patients with colonic NEC/MANEC from the SEER database according to the ENETS staging system (C), AJCC 8th staging system (D). Discrepancy between the numbers of patients at risk and the total size of the analytic cohort for each staging system is accounted for by the number of patients who were lost to follow‐up within 30 days. AJCC, American Joint Committee on Cancer; ENETS, European Neuroendocrine Tumor Society; MANEC, Mixed adeno‐neuroendocrine carcinoma; NEC, neuroendocrine carcinoma; NET, neuroendocrine tumor; SEER, Surveillance, Epidemiology, and End Results

Since the guidelines for poorly differentiated colonic NEC/MANEC in the ENETS and AJCC 8th staging systems were different, a TNM stage was assigned to each patient with colonic NEC/MANEC according to each system (Table 4). In Chinese patients, 125 patients had colonic NEC/MANEC, whose median follow‐up time was 0.98 years (range, 0.04‐8.97 years). The 1‐year DSS rates for stages I‐IV according to the ENETS criteria and the AJCC 8th criteria were 100%, 90.0%, 82.8% and 53.9% and 50.0%, 94.1%, 78.1%, and 53.9% respectively. According to both classifications, the survival comparisons were statistically significant when comparing stages (*P* < 0.001; Figure 2A,B). We further compared the predictive ability of the two staging systems by calculating the C‐index. The results showed that the AJCC staging system (0.673; 95% CI, 0.607‐0.739) had parallel prognostic efficacy to that of the ENETS system (0.660; 95% CI, 0.591‐0.729; *P* = 0.278).

**Table 4:** Stage of colonic NEC/MANEC based on the ENETS and AJCC 8th staging systems

Factor	Chinese cohort (N = 125)		SEER cohort (N = 267)	
	ENETS	AJCC 8th	ENETS	AJCC 8th
T classification^a^				
T1	3 (2.9%)	3 (2.9%)	4 (2.0%)	23 (10.4%)
T2	1 (1.0%)	1 (1.0%)	12 (5.9%)	11 (5.0%)
T3	55 (52.4%)	55 (52.4%)	110 (53.9%)	110 (49.5%)
T4	46 (43.8%)	46 (43.8%)	78 (38.2%)	78 (35.1%)
N classification^b^				
N0	37 (30.8%)	37 (30.8%)	51 (20.6%)	51 (20.6%)
N1	83 (69.2%)	—	197 (79.4%)	43 (17.3%)
N2	—	—	—	124 (50.0%)
N NOS	—	83 (69.2%)	—	30 (12.1%)
M classification^c^				
M0	58 (47.2%)	58 (47.2%)	106 (40.5%)	106 (40.5%)
M1	65 (52.8%)	65 (52.8%)	156 (59.5%)	156 (59.5%)
TNM stage^d^				
I	2 (1.7%)	3 (2.5%)	2 (0.8%)	5 (1.9%)
II	11 (9.2%)	20 (16.8%)	18 (7.0%)	27 (10.5%)
III	41 (34.5%)	31 (26.1%)	80 (31.3%)	69 (26.8%)
IV	65 (54.6%)	65 (54.6%)	156 (60.9%)	156 (60.7%)

Abbreviation: AJCC, American Joint Committee on Cancer; ENETS, European Neuroendocrine Tumor Society; MANEC, Mixed adeno‐neuroendocrine carcinoma; NEC, neuroendocrine carcinoma; NOS, Not otherwise specified; SEER, Surveillance, Epidemiology, and End Results.

a Data were available for 105 Chinese patients for both ENETS and AJCC 8th staging systems and 204 patients for ENETS staging and 222 patients for AJCC 8th staging from the SEER database.

b Data were available for 120 Chinese patients and 248 patients from the SEER database.

c Data were available for 123 Chinese patients and 262 patients from the SEER database.

d Data were available for 119 Chinese patients for both ENETS and AJCC 8th staging and 256 patients for ENETS staging and 257 patients for AJCC 8th staging from the SEER database.

We further performed analyses for validation in the SEER cohort, and similar results were found. A total of 267 patients had colonic NEC/MANEC in the SEER cohort, and the median follow‐up time was 0.71 years (range, 0.08‐13.33 years). The 1‐year DSS rates for stages I‐IV according to the ENETS criteria and the AJCC 8th criteria were 0, 100%, 66.6%, and 25.7% and 66.7%, 95.7%, 62.8%, and 25.7% respectively. Based on both classifications, the survival comparisons were statistically significant when comparing stages (*P* < 0.001, Figure 2C,D). The AJCC 8th system (0.670; 95% CI, 0.632‐0.708) did not show significantly better prognostic efficacy than the ENETS system (0.665; 95% CI, 0.627‐0.703; *P* = 0.063).

## 4 DISCUSSION

In the current study, we collected and analyzed the clinicopathological features of 167 colonic NENs from Chinese multicentric data and we also included 1248 patients from the SEER database for validation. Patients’ characteristics at baseline in the SEER cohort were significantly different from those of the Chinese cohort, which may be related to different races, diets and lifestyles. However, the clinical features of colonic NENs in the midgut and hindgut, and comparison of the prognostic efficacy of the ENETS and AJCC 8th staging systems were similar in the two cohorts, which may partly reflect the reliability of the results in the current study.

In colonic adenocarcinoma, an increasing number of studies have shown that tumors located in the left‐sided colon and the right‐sided colon, which originate from the hindgut and midgut, respectively, share different molecular features, characteristics, and prognoses as well as different responses to certain kinds of therapy, such as chemotherapy and EGFR antibody therapy.^17^, ^18^, ^19^, ^20^ However, a comparison of colonic NENs originating from the midgut and hindgut has not been reported.^12^, ^21^ Our study was the first to suggest that the two sites of colonic NENs were indeed different. One of the most distinct differences was that NEC/MANEC was much more common in colonic NENs in the midgut than in those in the hindgut. The current study showed that colonic NENs with different embryonic origins shared distinct pathological features, which reflected the high heterogeneity of NENs and may be related to various genetic and molecular alterations in different embryonic origins, which may result in a different response to certain drugs; this needs to be further studied in the future.

The only difference between the ENETS and AJCC 8th staging systems is the principles applied for colonic NEC/MANEC. In the ENETS staging system, the guidelines used for colonic NEC/MANEC are the same as those used for colonic NET, while in the AJCC 8th staging system, colonic NEC/MANEC is staged according to the principles used for colonic adenocarcinoma. Unlike the ENETS staging system, the AJCC 8th staging system only incorporates tumor invasion depth but not tumor size in the T definitions and classifies T2N0M0 as stage I and T4N0M0 as stage II (Appendices). However, our results showed that this modification did not improve the prognostic ability of the AJCC 8th staging system compared with the ENETS staging system with statistical significance in neither the SEER nor the Chinese cohorts. In view of the highly malignant biological behavior of colonic NEC/MANEC and the current low early tumor detection rate, more than 80% of patients with colonic NEC/MANEC were diagnosed with lymph nodes and distant metastases at the first visit, so a small proportion of patients had stage I/II disease according to both the ENETS and AJCC 8th criteria in SEER and Chinese cohorts. In addition, the *P* value of comparing the C‐index between the ENETS and AJCC 8th criteria in the SEER cohort was 0.063, which was close to the arbitrary cut off of 0.05. Thus, we should be cautious about drawing conclusions, and the early tumor detection rate should be improved while further investigating the performance of the staging systems. A staging system for colonic NENs based on the SEER database proposed by Landry et al was shown to have similar prognostic performance to the ENETS and AJCC 7th staging systems in colonic NENs.^22^, ^23^ However, this SEER‐based system defines the T stage in a much more complicated and less practical way compared with the ENETS and AJCC staging systems: while the SEER‐based system incorporates tumor size at each level of tumor invasion, the ENETS and AJCC 7th staging systems incorporate size only for T1 and T2, and the AJCC 8th system only incorporates tumor invasion for T definition. Therefore, the SEER‐based staging system has been less applied in clinical practice, and we did not incorporate this staging system for analyses in the current study.

As an uncommon kind of tumor, it is challenging to gather large numbers of patients with sufficient clinical data, and there were several limitations in our study. First, our study was a retrospective but not prospective study. However, we included 167 Chinese patients with colonic NENs from twelve hospitals in the current study, which is the largest series from Asia. We also included a large sample of patients with colonic NENs from the SEER database for validation. Second, the numbers of patients with well/moderately differentiated colonic NET in the Chinese cohort and of patients with colonic NEC/MANEC with stages I/II in both cohorts were small. Thus, the observed lack of proper interstage discriminatory power may represent type II errors. Hence, more studies are needed to verify our results. Finally, in the Chinese patients with colonic NEC/MANEC, the exact N stage could not be determined for all node positive patients with regard to the AJCC 8th staging system, and in both cohorts, the exact M stage could not be determined for all patients with distant metastasis according to the AJCC 8th staging system. Thus, we were unable to compare the prognostic efficacy of the two staging systems more specifically and accurately in the current study.

## 5 CONCLUSIONS

Our study showed that tumors originating from the midgut and the hindgut shared different clinicopathological features. The AJCC 8th staging system and the ENETS system appeared to have similar prognostic ability for colonic NEC/MANEC.

## CONFLICT OF INTEREST

None declared.

## APPENDIX 1

The ENETS staging definitions for colonic neuroendocrine neoplasms (NENs) and AJCC 8th staging definitions for colonic neuroendocrine carcinoma (NEC)^13^, ^15^

**Table:**

ENETS staging classification		AJCC 8th staging classification	
T1	Tumor invades mucosa or submucosa	T1	Tumor invades the submucosa (through the muscularis mucosa but not into the muscularis propria)
	T1a size <1 cm		
	T1b size 1–2 cm		
T2	Tumor invades muscularis propria or size >2 cm	T2	Tumor invades the muscularis propria
T3	Tumor invades subserosa/pericolic fat	T3	Tumor invades through the muscularis propria into the pericolic tissues
T4	Tumor directly invades other organs/structures and/or perforates visceral peritoneum	T4	Tumor invades the visceral peritoneum or invades or adheres to adjacent organ or structure
			T4a Tumor invades through the visceral peritoneum (including gross perforation of the bowel through tumor and continuous invasion of tumor through areas of inflammation to the surface of the visceral peritoneum)
			T4b Tumor directly invades or adheres to adjacent organs or structures
N0	No regional lymph node metastasis	N0	No regional lymph node metastasis
N1	Regional lymph node metastasis	N1	One to three regional lymph nodes are positive (tumor in lymph nodes measuring ≥0.2 mm), or any number of tumor deposits are present and all identifiable lymph nodes are negative
			N1a—One regional lymph nodes is positive
			N1b—Two or three regional lymph nodes are positive
			N1c—No regional lymph nodes are positive, but there are tumor deposits in the
			Subserosa
			Mesentery
			Or nonperitonealized pericolic tissues
		N2	Four or more regional nodes are positive
			N2a—Four to six regional lymph nodes are positive
			N2b—Seven or more regional lymph nodes are positive
M0	No distant metastasis	M0	No distant metastasis by imaging, etc.; no evidence of tumor in distant sites or organs
M1	Distant metastasis	M1	Metastasis to one or more distant sites or organs or peritoneal metastasis is identified
			M1a—Metastasis to one site or organ is identified without peritoneal metastasis
			M1b—Metastasis to two or more sites or organs is identified without peritoneal metastasis
			M1c—Metastasis to the peritoneal surface is identified alone or with other site or organ metastases

**Table:**

Stage				Stage			
IA	T1a	N0	M0	I	T1, T2	N0	M0
IB	T1b	N0	M0	IIA	T3	N0	M0
IIA	T2	N0	M0	IIB	T4a	N0	M0
IIB	T3	N0	M0	IIC	T4b	N0	M0
IIIA	T4	N0	M0	IIIA	T1‐T2	N1/N1c	M0
					T1	N2a	M0
IIIB	Any T	N1	M0	IIIB	T3‐T4a	N1/N1c	M0
					T2‐T3	N2a	M0
					T1‐T2	N2b	M0
				IIIC	T4a	N2a	M0
					T3‐T4a	N2b	M0
					T4b	N1‐N2	M0
IV	Any T	Any N	M1	IVA	Any T	Any N	M1a
				IVB	Any T	Any N	M1b
				IVC	Any T	Any N	M1c
